# Rice (*Oryza sativa* L.) bran preserves cardiac function by modulating pro-inflammatory cytokines and redox state in the myocardium from obese rats

**DOI:** 10.1007/s00394-021-02691-0

**Published:** 2021-10-12

**Authors:** Jéssica Leite Garcia, Danielle Fernandes Vileigas, Cristina Schmitt Gregolin, Mariane Róvero Costa, Fabiane Valentini Francisqueti-Ferron, Artur Junio Togneri Ferron, Dijon Henrique Salomé De Campos, Fernando Moreto, Igor Otávio Minatel, Silméia Garcia Zanati Bazan, Camila Renata Corrêa

**Affiliations:** 1 São Paulo State University (UNESP), Medical School, Botucatu, Brazil; 2 Department of Biochemistry, Institute of Chemistry, University of São Paulo (USP), São Paulo, Brazil; 3 São Paulo State University (UNESP), Institute of Biosciences, Botucatu, Brazil

**Keywords:** Rice bran, Western diet, Inflammation, Oxidative stress, Cardiac remodeling, Insulin resistance

## Abstract

**Purpose:**

This study aimed to evaluate the effect of rice bran (RB) supplementation to a high-sugar fat (HSF) diet on cardiac dysfunction in an experimental obesity model.

**Methods:**

Male Wistar rats were distributed into three groups: control, high-sugar fat, and high-sugar fat supplemented with 11% RB for 20 weeks.

**Results:**

HSF diet promoted obesity and metabolic complications. Obese rats showed cardiac structural and functional impairment associated with high levels of interleukin-6, tumoral necrosis factor alpha, and malondialdehyde, and decreased activity of superoxide dismutase and catalase in the myocardium. RB supplementation was able to mitigate obesity and its metabolic alterations in HSF diet-fed animals. Moreover, the RB also prevented structural and functional damage, inflammation, and redox imbalance in the heart of these animals.

**Conclusion:**

This study suggests that RB supplementation prevents cardiac dysfunction in rats fed on HSF by modulating systemic metabolic complications and inflammation and oxidative stress in the myocardium, representing potential alternative therapy.

## Introduction

The contemporary dietary habits, especially the high consumption of processed foods rich in sugar and fat, associated with overnutrition and a sedentary lifestyle, have significantly increased the worldwide prevalence of obesity [1, 2], a complex metabolic disease defined as an abnormal or excessive accumulation of adipose tissue within the body that may impair health [3].

A consistent body of evidence shows that obesity promotes hemodynamics, neurohormonal and metabolic changes that may contribute to alterations in cardiac structure and ventricular function, progressing to heart failure depending on the duration and severity of obesity [4–6]. The pathophysiology of cardiac dysfunction due to excess adipose tissue is complex. In addition to metabolic complications such as dyslipidemia, insulin resistance, hyperglycemia, and hyperinsulinemia, the quality per ser of the diet can also cause an attack on the heart by different mechanisms [1, 6]. Evidence suggests that the imbalance of the redox state, also known as oxidative stress, and the high release of pro-inflammatory cytokines are strongly involved in the etiology of obesity-related cardiomyopathy. Thus, to investigate therapeutic strategies is essential for the prevention and treatment of heart disease owing to obesity [7, 8].

The consumption of plant foods, rich in an array of bioactive compounds, has emerged as an alternative to ameliorate the harmful effects of obesity and its related disorders [9, 10]. Rice (*Oryza sativa L.*) is the second most-consumed grain in the world, after wheat, and constitutes the dietary stable of half of the world population [11, 12]. Rice is preferably consumed in the polished form (white rice); however, the bran layer removed from the rice contains the major bioactive compounds [13]. Rice bran (RB) is a byproduct of the rice milling industry, obtained after rice kernel polishing. It is noteworthy that, every year, around 90% of RB is used as animal feed or discarded [11]. RB constitutes about 10% of the total weight of rough rice and contains hundreds of different bioactive components, mainly tocopherols, tocotrienols, and oryzanols, and in less concentration, carotenoids, lecithin, and long-chain alcohols. The γ-oryzanol and vitamin E are the main antioxidants in RB, and the γ-oryzanol levels are around 20 times higher than vitamin E, varying according to the growing environment and rice genotypes [14, 15].

In the past few years, RB has gained attention for its hypocholesterolemic, anti-diabetic, anti-obesogenic, antioxidant, and anti-inflammatory effects, mitigating the risk factors for cardiovascular diseases. Such effects have been attributed mainly to γ-oryzanol [13, 16–19]. RB shows vast potential due to beneficial health effects, nutritional value, low cost, and easy availability, despite the RB is still under-used food for human consumption and poorly explored. Moreover, it can be readily incorporated into the diet as a food supplement, representing a natural therapeutic alternative that can avoid undesirable side effects compared to pharmaceutical drugs [11, 14].

Although the beneficial impact on health is well known, its effects on mitigating cardiac dysfunction have not yet been evaluated in obesity. Therefore, this study aimed to evaluate the effect of RB supplementation to a high-sugar fat on cardiac dysfunction in an experimental obesity model.

## Methods

### Animals and experimental design

Male Wistar rats (± 325 g) obtained from the Animal Center of Botucatu Medical School, Sao Paulo State University (UNESP, Botucatu, SP, Brazil) were randomly distributed into three groups (*n* = 8 per group): control diet (C), high-sugar fat diet (HSF), and high-sugar fat diet supplemented with RB (HSF + RB) for 20 weeks. HSF groups also received 25% sucrose in drinking water, whereas normal drinking water without any supplementation was given to C rats. The animals were housed in individual cages under controlled temperature (22 ± 3 ◦C), luminosity (12 h light/dark cycle) and humidity (60 ± 5%), and received diets and water ad libitum.

At the end of the experimental period, following the echocardiogram and systolic blood pressure evaluation, the animals were fasted for 8 h and then anesthetized (thiopental 120 mg/kg/i.p.) and euthanized by decapitation after verification of the absence of foot reflex. Blood samples were collected, and the plasma was separated by centrifugation (800 × *g* at 4 °C for 10 min) for metabolic and hormonal analyzes. The adipose tissue was isolated, dissected, and weighed for nutritional profile assessment. Fresh feces and cardiac tissue were also collected from each animal for further analysis.

The study was performed according to the guidelines for animal research [20] and approved by the Ethics Committee on Animal Experiments of the Botucatu Medical School, UNESP (protocol number 1305/2019).

### Diet composition

All the diets used in this experiment were nutritionally balanced for micronutrients but different for macronutrients. The ingredients and nutritional composition of the diets are presented in Table 1. The C and HSF diets were previously described [21–23]. RB was gently provided by Brasília Alimentos Ltda (Santa Cruz do Rio Pardo, São Paulo, Brazil) and obtained from *Oryza sativa L.* rice processing. The RB was heat-stabilized to inactivate the enzymes responsible for the degradation of its components and prevent rancidity before being mixed with the other ingredients for the diet preparations [11, 24]. The HSF + RB diet was customized containing 11% (wt/wt) of RB [25].

**Table 1:** Ingredients and nutritional composition of diets

Components (g/kg)	C	HSF	HSF + RB
Soybean meal	335	340	340
Sorghum	278	80	80
Soy hulls	188	116	6
Dextrin	146	20	20
Sucrose	–	80	80
Fructose	–	180	180
Soybean oil	23	–	–
Lard	–	154	154
Rice bran	–	–	110
Vitamin and mineral premix	25	25	25
Salt	4	8	8

Nutritional values			
Carbohydrate (% of ingredients)	60.0	53.5*	55.1*
Protein (% of ingredients)	20.0	18.0	18.1
Fat (% of ingredients)	4.00	16.5	18.3
UFA	69.0	47.0	62.0
SFA	31.0	53.0	38.0
Others (%)**	16.0	12.0	8.50
Carbohydrates (% calories)	66.8	49.2*	48.3*
Protein (% calories)	22.9	16.6	15.8
Fat (% calories)	10.4	34.2	35.9
Energy (Kcal/g)	3.59	4.35*	4.58*

*C* control diet; *HSF* high-sugar fat diet; *RB* rice bran; *UFA* unsaturated fatty acids; *SFA* saturated fatty acids

*% of carbohydrate and energy in chow without considering the sucrose added in drinking water

**Ashes and fibers

### Nutritional profile

The nutritional profile of the animals was assessed according to the following parameters: food and caloric intake, water intake, body weight, body fat, adiposity index, and fecal fat. Food and water intake were daily calculated from the individual leftovers of each animal, and the caloric intake was determined by multiplying the energy value of each diet (*g* × Kcal) by the daily food consumption. For the groups fed the HSF diet, the caloric intake also included the calories from the sucrose in drinking water. The body weight was measured weekly. The body fat was determined by the sum of epididymal, retroperitoneal, and visceral fat pad weights. The adiposity index was calculated as follows: [body fat (g)/final weight (g)] × 100. The fecal fat content was assessed by the acid steatocrit method [26]. Briefly, random spots from the fresh feces were collected, and 0.5 g was diluted in water, homogenized, and then mixed with perchloric acid 5 M. The mixture was aspirated into a capillary tube, and one end was sealed and centrifuged in a centrifuge for capillary tubes (10,000 × *g*, 15 min). The fat and solid layers were measured, and the acid steatocrit was calculated by the formula: fat layer/(fat layer + solid layer) × 100 [27].

### Metabolic and hormonal analysis in plasma

Triglycerides concentrations were measured using specific kits (BIOCLIN^®^, Belo Horizonte, MG, Brazil) and analyzed by a colorimetric-enzymatic method in an automatic enzymatic analyzer system (Chemistry Analyzer BS-200, MindrayMedical International Limited, Shenzhen, China). The glucose level was measured in a blood drop using a handheld glucometer (Accu-Chek Performa, Roche Diagnostics Brazil Limited, SP, Brazil). The insulin levels were evaluated by an enzyme-linked immunosorbent assay (ELISA) method (EMD Millipore Corporation, Billerica, MA, USA), according to the manufacturer’s instructions, and the reading was recorded using a microplate reader (Spectra Max 190, Molecular Devices^®^, Sunnyvale, CA, USA). Insulin resistance was estimated according to the homeostatic model assessment for insulin resistance (HOMA-IR) index using the following formula: fasting insulin (μU/mL) × fasting glucose (mmol/L)/22.5.

### Cardiovascular profile

#### Systolic blood pressure

The systolic blood pressure (SBP) analysis was assessed in conscious rats by a non-invasive tail-cuff method with a NarcoBioSystems^®^ Electro-Sphygmomanometer (International Biomedical, Austin, TX, USA). The animals were warmed in a wooden box (50 × 40 cm) between 38 and 40 °C for 4 min to stimulate arterial vasodilation. After this procedure, a cuff with a pneumatic pulse sensor was attached to the tail of the animal. The cuff was inflated to 200 mmHg pressure and subsequently deflated. The arterial pulsations were recorded in a computerized data acquisition system (AcqKnowledge^®^ MP100, Biopac Systems Inc., Santa Barbara, CA, USA). The average of three pressure readings was obtained for each animal [28].

#### Echocardiographic study

Doppler echocardiographic evaluation was performed by a single examiner using commercially available echocardiography (General Electric Medical Systems, Vivid S6, Tirat Carmel, Israel) equipped with a 5–11.5 MHz multifrequency ultrasonic transducer. Animals were anesthetized via intraperitoneal injection of a mixture of ketamine (50 mg/kg) and xylazine hydrochloride (1 mg/kg). After trichotomy of the anterior chest region, the animals were placed in slight left lateral decubitus for the exam. For structural measurements of the heart, the images were obtained in one-dimensional mode (M-mode) guided by the images in two-dimensional mode with the transducer in the parasternal position, minor axis. Left ventricular (LV) evaluation was performed by positioning the cursor M-mode just below the mitral valve plane at the papillary muscles level. The images of the aorta and left atrium were obtained by positioning the M-mode course to plan the aortic valve level.

The following LV structural parameters were analyzed: diameters of the left atrium (LA) and aorta (AO), LV diastolic diameter (LVDD), and LV relative wall thickness (RWT). The LV systolic function was assessed by ejection fraction (EF), posterior wall shortening velocity (PWSV), and tissue Doppler imaging (TDI) of mitral annulus systolic velocity (S’). The LV diastolic function was evaluated by early and late diastolic mitral inflow velocities (E and A waves), E/A ratio, E wave deceleration time (EDT), TDI of early (E’) and late (A’) mitral annulus diastolic velocity (average of septal and lateral walls), and E’/A’ and E/E’ ratios [29].

#### Inflammation and redox state in myocardium

##### Tissue preparation

The LV samples were homogenized in phosphate-buffered saline (PBS) pH 7.4 (1:10; w/v) using a bead beater homogenizer (Bullet Blender^®^, Next Advance, Inc., NY, USA). The homogenates were centrifuged at 800 × *g* for 10 min at 4 °C (Eppendorf^®^ Centrifuge 5804-R, Hamburg, Germany), and the supernatant was obtained to measure the pro-inflammatory cytokines and redox state, such as antioxidant enzymes activity and oxidative markers. All the analyses were normalized by the total protein content, which was determined using a colorimetric method (BioClin, Quibasa Química Básica Ltda., Belo Horizonte, MG, Brazil), and the readings were performed in a microplate reader.

##### Pro-inflammatory cytokines

Interleukin-6 (IL-6) and tumoral necrosis factor alpha (TNF-α) were assessed by ELISA assay (EMD Millipore Corporation, Billerica, MA, USA), according to manufactures’ instructions, and the readings were performed in a microplate reader.

##### Antioxidant enzymes activity

Superoxide dismutase (SOD) activity was measured based on the inhibition of superoxide radical reaction with pyrogallol by spectrophotometry at 420 nm. One unity of SOD activity (U) is defined as the quantify of the enzyme that inhibited 50% of pyrogallol autoxidation, and the results were expressed in U/mg protein/minute [30].

Catalase (CAT) activity was evaluated by the decrease in hydrogen peroxide (H_2_O_2_) levels. The breakdown of H_2_O_2_ in the reaction mixture was measured spectrophotometrically at 240 nm, and the results were expressed as pmol/mg protein/minute [31].

Glutathione peroxidase (GPx) activity was assessed spectrophotometrically at 340 nm by following β-nicotinamide adenine dinucleotide phosphate (NADPH) oxidation as described by Flohé and Günzler (1984) [32]. The enzymatic activity was expressed as μmol/mg protein/minute.

##### Oxidative stress markers

Protein carbonylation (CBO) was measured in the supernatant by a photometric assay using DNPH (2,4-dinitrophenyl hydrazine) as derivatizing agent [33]. All the procedures were performed protected from light. The supernatant previously prepared was diluted (1:10), and then 10 µl was incubated with 100 µl of 10 mM DNPH solution for 10 min. Following this, 50 µl of 6 M NaOH (sodium hydroxide) was added and incubated for 10 min. Absorbance was recorded spectrophotometrically at 450 nm. The results were calculated with molar extinction coefficient (22,000 M^−1^ cm^−1^) of DNPH, and expressed as nmol/mg protein.

Malondialdehyde (MDA) concentrations were measured to estimate lipid peroxidation levels. The samples were mixed with 10% trichloroacetic acid (TCA) for protein precipitation in the proportion 1:4 (sample: TCA). After centrifugation (800 × *g*, 5 min), the supernatant was removed, and 0,67% thiobarbituric acid (TBA) was added (1:1). Then, the samples were heated for 15 min at 100 °C, and after cooling, the absorbance readings were acquired using spectrophotometer at 535 nm. The MDA concentration was obtained by the molar extinction coefficient (1.56 × 10^5^ M^−1^ cm^−1^), and expressed in nmol/mg protein [34].

### Statistical analysis

The data were tested for normality using the Shapiro–Wilk test and for homogeneity of variance using Levene’s test prior to statistical analysis. The data were compared by one-way ANOVA followed by Tukey post-hoc test for parametric data or Kruskal–Wallis followed by Dunn’s post-hoc test for non-parametric data. Data were expressed as mean ± standard deviation (SD) or median {minimum [min]–maximum [max]}.

All numerical biological results were analyzed using a web tool for visualizing the clustering of multivariate data ClustVis [35] for exploring the unsupervised multivariate principal component analysis (PCA) and hierarchical clustering analyses. The correlation matrix with all data was analyzed using a 2-tailed Pearson’s correlation test.

The analyses were performed using SigmaPlot 12.0 for Windows (Systat Software Inc., San Jose, CA, USA). All graphics were generated using GraphPad Prism 8 (GraphPad Software Inc., San Diego, CA, USA). The differences were considered statistically significant when *p* < 0.05.

## Results

### Rice bran supplementation prevented weight gain and increased fecal fat

**Fig. 1:**
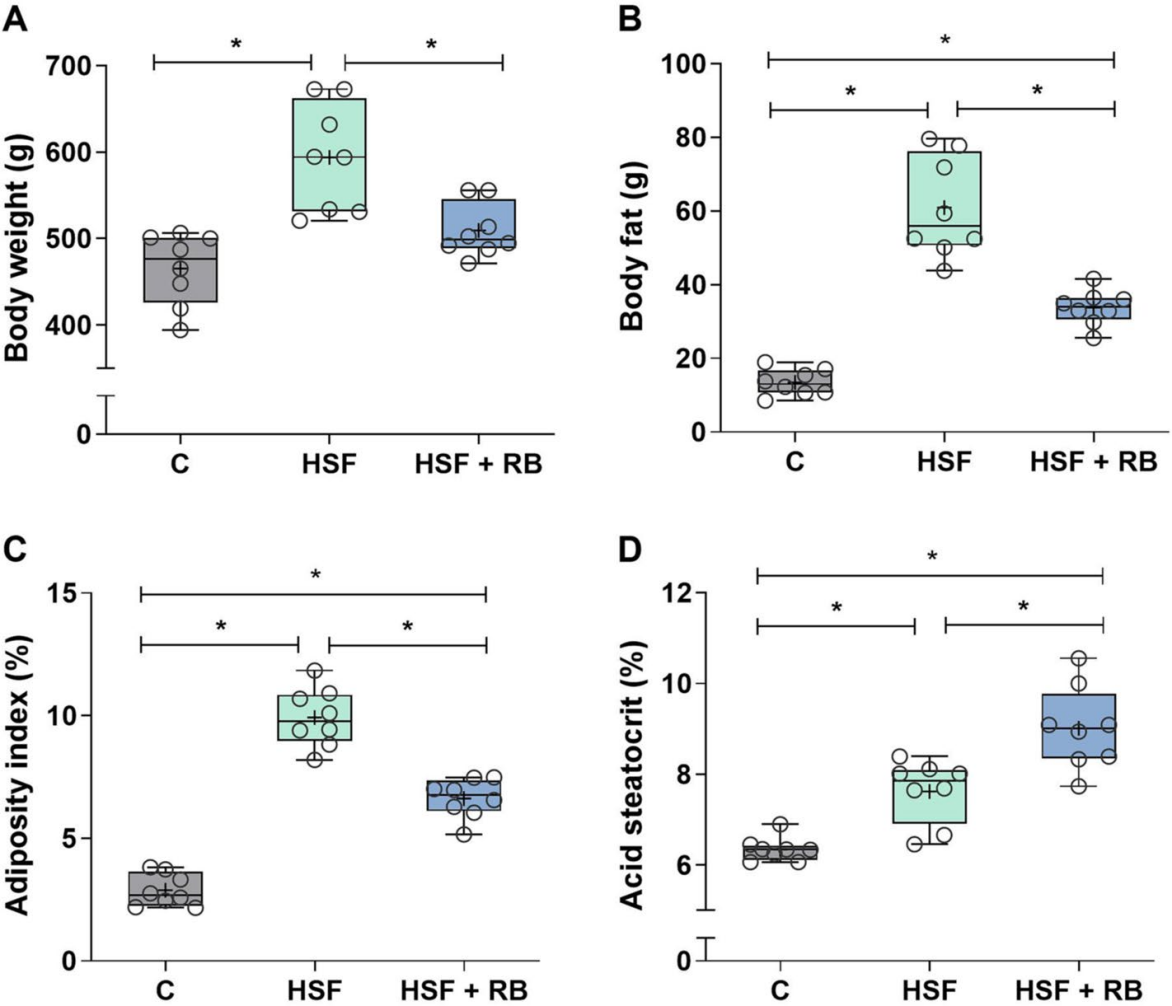
Nutritional profile. (**A**) Body weight, **(B**) body fat, (**C**) adiposity index, and (**D**) acid steatocrit of the control diet (C), high-sugar fat diet (HSF), and high-sugar fat diet supplemented with rice bran (HSF + RB) groups. Data expressed as median (min—max). Mean is indicated as + . *p < 0.05. ANOVA followed by Tukey for **A**, **B**, **C** and **D**

Body weight gain mainly depends on food and calorie intake, and such information provides insights into the progression of obesity. The HSF and HSF + RB groups presented a decrease in food intake accompanied by increased water and calorie intake compared to the C group. In addition, there is no interference of RB in food, water, and calorie intake since these parameters were similar between HSF and HSF + RB groups (Table 2). Prolonged exposure to HSF caused a significant rise in body weight, body fat, and adiposity index, leading the animals to obesity (Fig. 1A–C). RB was able to prevent the weight gain in the rats fed HSF since there is no difference in body weight between HSF + RB and C groups (Fig. 1A). Although the body fat and adiposity index remained higher in the HSF + RB animals compared to the C group, these parameters showed a significant decrease, of 44.6% and 33.3%, respectively, in the rats fed HSF supplemented with RB in relation to animals under the HSF but without supplementation (Figs. 1B and C). The acid steatocrit values were statistically different comparing the three groups, and RB led to higher excretion of the fecal fat in the animals (Fig. 1D).

**Table 2:** Dietary intake of the control diet (C), high-sugar fat diet (HSF), and high-sugar fat diet supplemented with rice bran (HSF + RB) groups

Variables	C	HSF	HSF + RB
Food intake (g/day)	22.8 (20.3–24.2)	12.3 (11.9–12.8)*	11.0 (9.21–13.0)*
Water intake (mL/day)	32.4 ± 5.22	51.6 ± 8.07*	50.5 ± 7.93*
Caloric intake (kcal/day)	81.3 ± 4.82	105 ± 6.53*	101 ± 2.77*

Data expressed in mean ± SD or median (min–max) compared by one-way ANOVA followed by Tukey post-hoc or Kruskal Wallis followed by Dunn’s *post-hoc* test

*p* < 0.05: *vs C

### Insulin resistance and lipid profile changes are prevented by RB supplementation

**Fig. 2:**
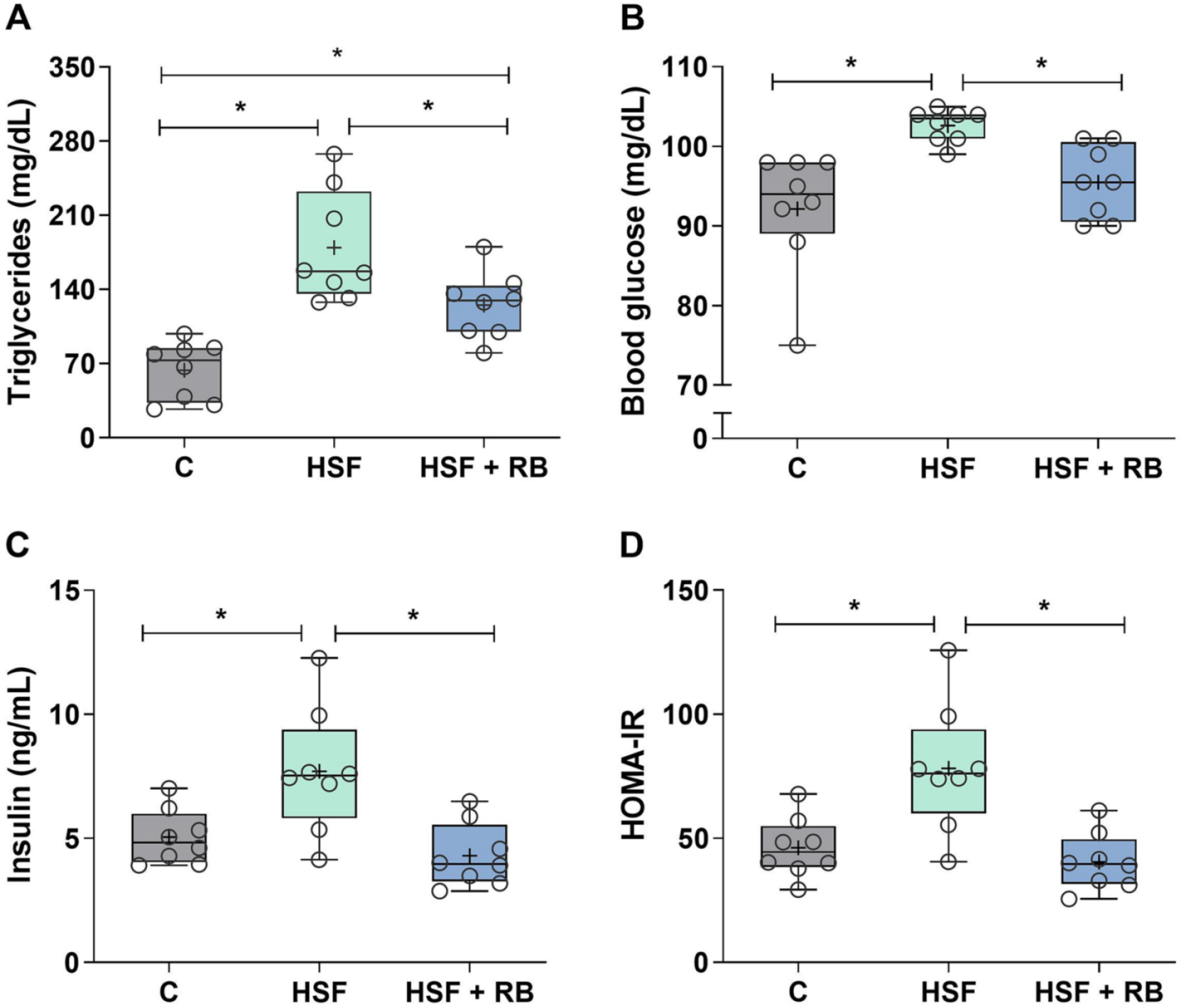
Plasma metabolic and hormonal analyses. (**A**) Triglycerides, (**B**) glucose, (**C**) insulin, and (**D**) homeostatic model assessment of insulin resistance (HOMA-IR) index of the control diet (**C**), high-sugar fat diet (HSF), and high-sugar fat diet supplemented with rice bran (HSF + RB) groups. Data expressed as median (min–max). Mean is indicated as + . *p < 0.05. ANOVA followed by Tukey for **A**, **C** and **D**; Kruskal–Wallis followed by Dunn’s for **B**

HSF diet-induced obesity promoted significant metabolic and hormonal changes (Fig. 2). The plasma triglycerides, glucose, and insulin levels and HOMA-IR were higher in HSF rats than in C rats (Fig. 2A–D). Although the plasma triglycerides remain elevated in the HSF + RB animals in relation to the C group, these values were 43.4% lower in HSF + RB group compared to the HSF group (Fig. 2A). Additionally, the elevation of glucose and insulin levels was prevented by RB supplementation in animals fed HSF since the values were similar to C group (Figs. 2B and C), as well as the insulin resistance, assessed by HOMA-IR (Fig. 2D), a marker of metabolic homeostasis.

### RB supplementation preserves cardiac structure and function in rats fed HSF

The SBP and cardiac structural and functional data, evaluated by echocardiogram, are shown in Table 3. The SBP was elevated in HSF and HSF + RB groups compared to the C group, and RB supplementation did not change this condition in relation to animals fed HSF diet without RB. HSF-induced obese rats presented structural changes (LA, LA/AO, LVDD, and RWT) and systolic (EF, PWSV, and S’ media) and diastolic (EDT, E/E’, and E’/A’) dysfunction compared to the C group. All these cardiac structural and functional parameters were preserved by RB supplementation in animals fed HSF, suggesting the potential of the RB in preventing cardiac disorders in the obesity model.

**Table 3:** Systolic blood pressure (SBP) and echocardiography study of the control diet (C), high-sugar fat diet (HSF), and high-sugar fat diet supplemented with rice bran (HSF + RB) groups

Variables	C	HSF	HSF + RB
SBP (mmHg)	123 ± 4.94	140 ± 9.27*	137 ± 5.02*
HR (bpm)	228 (208–271)	285 (118–369)	254 (150–312)
LA (mm)	4.60 (4.60–4.85)	5.75 (5.36–6.39)*	4.85 (4.70–4.90)^#^
AO (mm)	3.83 (3.58–3.83)	3.70 (3.32–4.34)	4.09 (3.58–4.09)
LA/AO	1.23 (1.20–1.35)	1.53 (1.23–1.77)*	1.19 (1.18–1.31)^#^
LVDD (mm)	7.49 ± 0.49	6.78 ± 0.33*	7.41 ± 0.44^#^
RWT	0.39 ± 0.00	0.55 ± 0.05*	0.41 ± 0.02^#^
EF (%)	0.93 ± 0.00	0.89 ± 0.02*	0.92 ± 0.01^#^
PWSV (cm/s)	74.5 ± 5.60	66.2 ± 6.83*	75.8 ± 5.59^#^
S’ media (cm/s)	5.48 ± 0.11	4.93 ± 0.19*	5.53 ± 0,25^#^
E wave (cm/s)	73.3 ± 8.37	81.7 ± 6.64	76.9 ± 3.78
E/A	1.74 ± 0.36	1.65 ± 0.40	1.89 ± 0.44
EDT (ms)	48.7 ± 3.53	58.3 ± 4.26*	48.8 ± 3.68^#^
E’/A’	1.62 (1.39–1.70)	0.63 (0.58–1.89)*	1.52 (1.29–1.81)^#^
E/E’	14.0 (11.1–15.5)	23.4 (28.2–16.8)*	14.1 (13.2–15.2)^#^

Data expressed in mean ± SD or median (min–max) compared by one-way ANOVA followed by Tukey post-hoc or Kruskal Wallis followed by Dunn’s post-hoc

*SBP* systolic blood pressure; *HR* heart rate; *LA* left atrium diameter; *AO* aorta diameter; *LVDD* left ventricle (LV) diastolic diameter; *RWT* LV relative wall thickness; *EF* ejection fraction; *PWSV* posterior wall shortening velocity; *S’* tissue Doppler imaging (TDI) of mitral annulus systolic velocity; *E* early diastolic mitral inflow velocity; *A* late diastolic mitral inflow velocity; *EDT* E wave deceleration time; *E’* TDI of early mitral annulus diastolic velocity; *A’* TDI of late mitral annulus diastolic velocity

*p* < 0.05: *vs C; ^#^vs HSF

### RB mitigated IL-6 and TNF-α levels in heart tissue

**Fig. 3:**
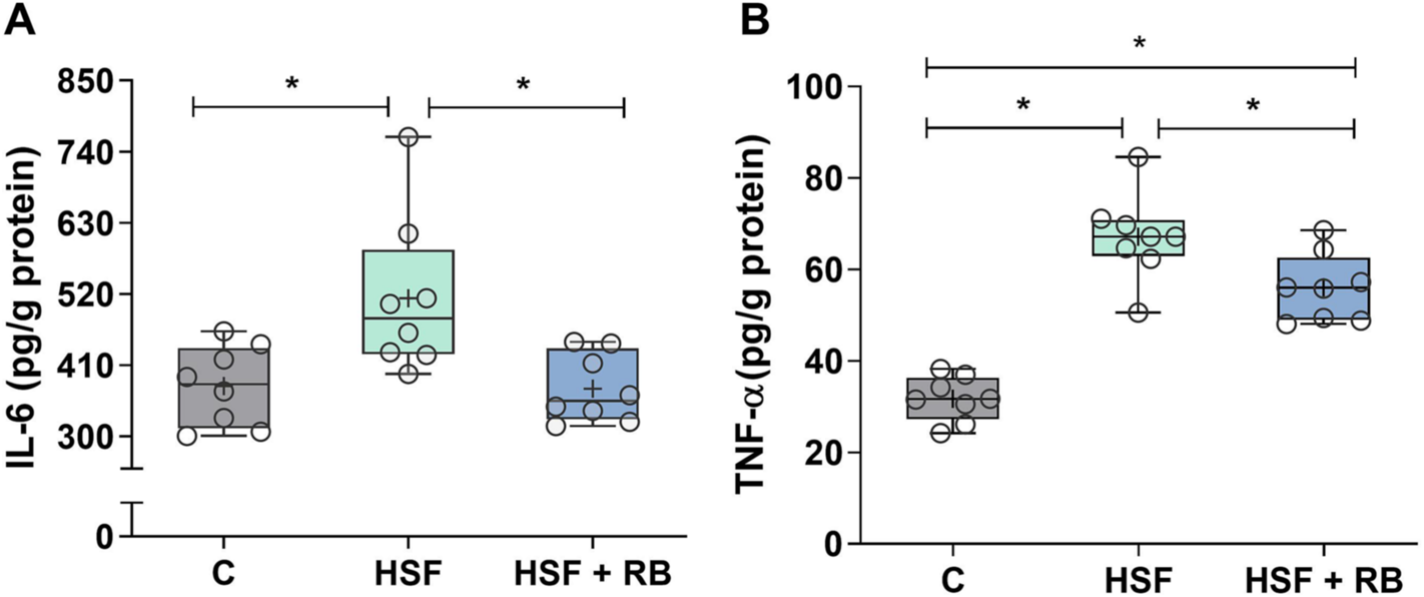
Pro-inflammatory cytokines in cardiac tissue. (**A**) Interleukin-6 (IL-6) and (**B**) tumoral necrosis factor alpha (TNF-α) levels of the control diet (**C**), high-sugar fat diet (HSF), and high-sugar fat diet supplemented with rice bran (HSF + RB) groups. Data expressed as median (min–max). Mean is indicated as + . *p < 0.05. ANOVA followed by Tukey for **B**; Kruskal–Wallis followed by Dunn’s for **A**

Inflammation is an important obesity-related disorder, and to verify the preventive effect of RB on this condition in a HSF-induced obesity model, we evaluated the pro-inflammatory cytokines, as IL-6 and TNF-α, in the heart tissue. The obesity due to the HSF diet caused cardiac inflammation, observed by elevated IL-6 and TNF-α cytokine (Fig. 3A and B). The RB supplementation was able to prevent the rise of IL-6 in rats fed HSF since the values were similar to the rats fed C diet (Fig. 3A). In contrast, the HSF + RB group showed higher levels of TNF-α compared to the C group, despite mitigating the elevation of this cytokine by presenting a lower concentration concerning the HSF group (Fig. 3B).

### RB supplementation prevents redox imbalance in heart tissue of HSF-fed rats

**Fig. 4:**
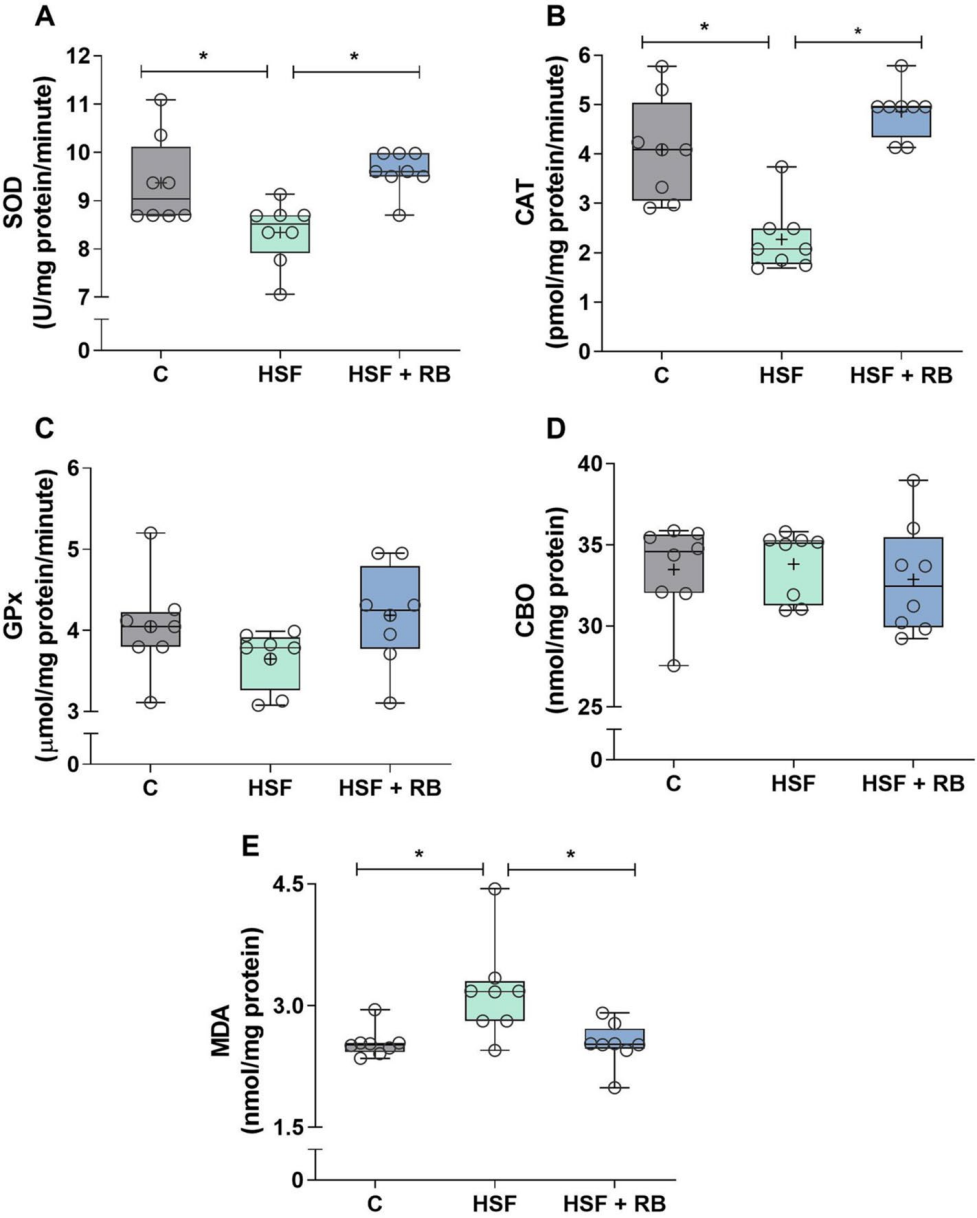
Antioxidant enzyme activities and oxidative markers in cardiac tissue. (**A**) Superoxide dismutase (SOD), (**B**) catalase (CAT), and (**C**) glutathione peroxidase (GPx) activities and (**D**) protein carbonylation (CBO) and (**E**) malondialdehyde (MDA) levels of the control diet (C), high-sugar fat diet (HSF), and high-sugar fat diet supplemented with rice bran (HSF + RB) groups. Data expressed as median (min–max). Mean is indicated as + . *p < 0.05. ANOVA followed by Tukey for **A**, **B**, **C** and **D**; Kruskal–Wallis followed by Dunn’s for **E**

The activity of antioxidant defense enzymes and pro-oxidant markers were measured to evaluate the preventive effect of RB in the cardiac redox state of the rats with HSF-induced obesity (Fig. 4). The antioxidant enzyme activities, SOD and CAT, were decreased in HSF group compared to the C animals. Both enzymes were significantly restored by RB supplementation in the HSF + BR group to similar levels of C rats. There was no difference in GPx activity comparing all groups. Concerning the oxidative stress markers, only the MDA levels were affected by obesity since the HSF-fed animals showed higher values than C rats. Moreover, the HSF + RB group was able to maintain this parameter at levels similar to the C group and lower than the HSF group.

### Global correlation map, multivariate and clustering analyses

**Fig. 5:**
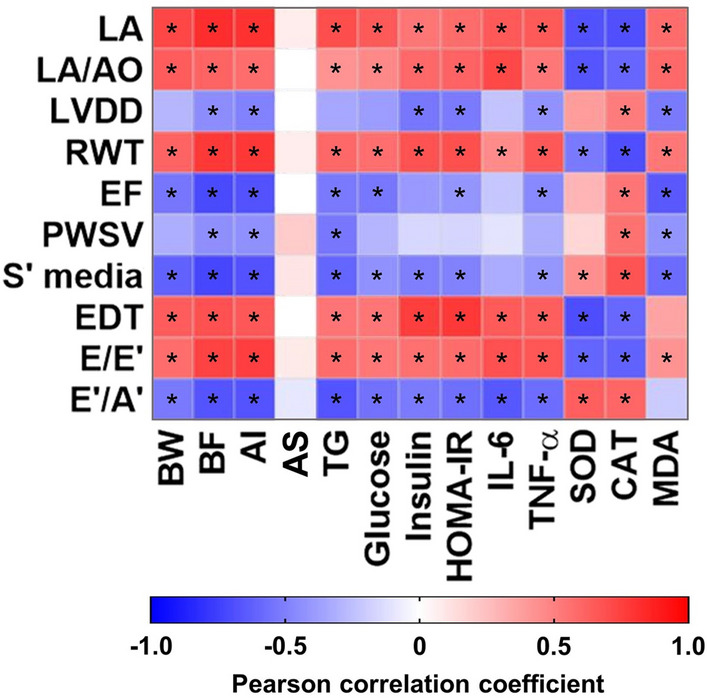
Correlation among structural and functional cardiac data and nutritional, metabolic, hormonal, inflammatory, and oxidative profiles visualized by a heatmap. *LA* left atrium diameter; *AO* aorta diameter; *LVDD* left ventricle (LV) diastolic diameter; *RWT* LV relative wall thickness; *EF* ejection fraction; *PWSV* posterior wall shortening velocity; *S’* tissue Doppler imaging (TDI) of mitral annulus systolic velocity; *EDT* E wave deceleration time; *E* early diastolic mitral inflow velocity; *E’* TDI of early mitral annulus diastolic velocity; *A’* TDI of late mitral annulus diastolic velocity; *BW* body weight; *BF* body fat; *AI* adiposity index; *AS* acid steatocrit; *TG* triglycerides; *IL-6* interleukin-6; *TNF-α* tumoral necrosis factor alpha; *SOD* superoxide dismutase; *CAT* catalase and *MDA* malondialdehyde. Data of all experimental groups were gathered and analyzed by GraphPad Prism 8 software using a 2-tailed Pearson’s correlation test. As shown by the color scale, the blue color indicates a negative correlation, while the red color indicates a positive correlation. The symbol * indicates a statistically significant correlation (*p* < 0.05)

A correlation matrix via Pearson’s correlation was performed to correlate cardiac structure and function parameters with other variables assessed (Fig. 5). The cardiac structural and functional variables were significantly correlated to body fat and adiposity index, negatively or positively, showing a more substantial influence of adiposity than body weight. As the body fat and adiposity index increases, the structure variables rise as well, except LVDD. The systolic function parameters decrease as the body fat and adiposity index increase. The acid steatocrit did not show a correlation to the cardiac parameters. The biochemical and metabolic variables, triglycerides, glucose, insulin, and HOMA-IR were correlated to most cardiac variables. Cardiac biomarkers of inflammation and oxidative stress were also associated with cardiac structure and function. IL-6 and TNF-a demonstrated a positive correlation with LA, LA/AO, RWT, EDT, and E/E’ and were inversely related to E’/A’. Moreover, TNF-α was also inversely related to LVDD, EF, and S’ media. The antioxidant enzymes, SOD and CAT, presented an inverse correlation to the other parameters, as expected since the enzymes act in the opposite direction preventing the cardiac changes. CAT showed significance to all cardiac parameters.

**Fig. 6:**
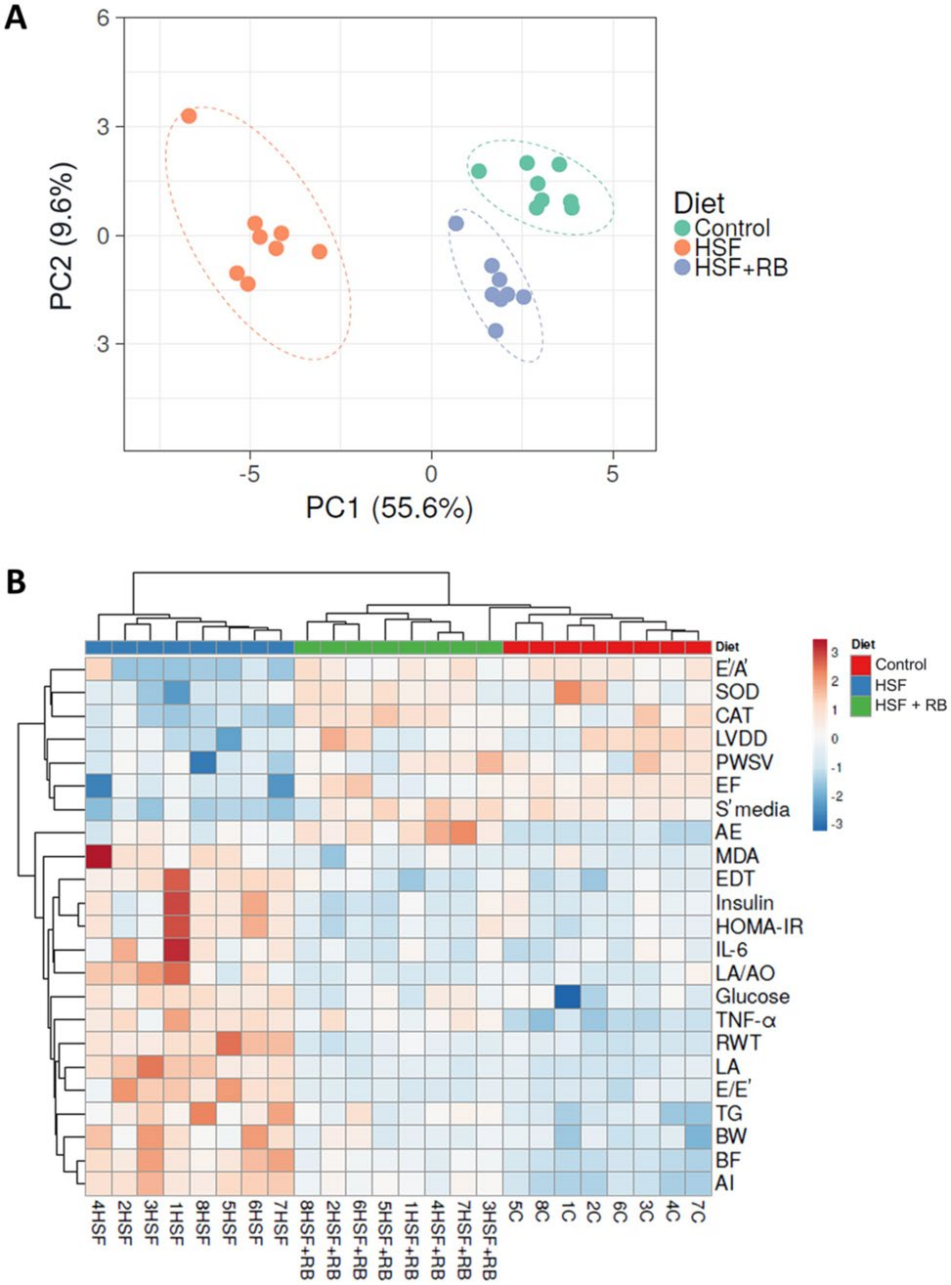
(**A**) Unsupervised multivariate principal component analysis (PCA) plot showing the multivariate variation among control, high-sugar fat (HSF), and high-sugar fat supplemented with rice bran (HSF + RB) diets in relation to all evaluated parameters. Each data point represents a sample, which is colored by the annotation of its diet type. (**B**) Hierarchical clustering analyses (Heatmap) using unsupervised Euclidean distance summarizes the effect of rice bran (RB) on the studied parameters of rats fed on a high-sugar fat diet (HSF). All evaluated parameters abbreviations are as defined in Fig. 5

Unsupervised multivariate principal component analysis (PCA) (Fig. 6A) and hierarchical cluster analysis (Fig. 6B) indicates a very well separation of the groups. In both analysis, HSF group is clustered away from C group indicating the efficiency of the model to induce the changes. The HSF + RB group is placed between the other two groups, closer to C group than HSF, indicating the beneficial effect of RB to prevent the changes induced in HSF group.

## Discussion

**Fig. 7:**
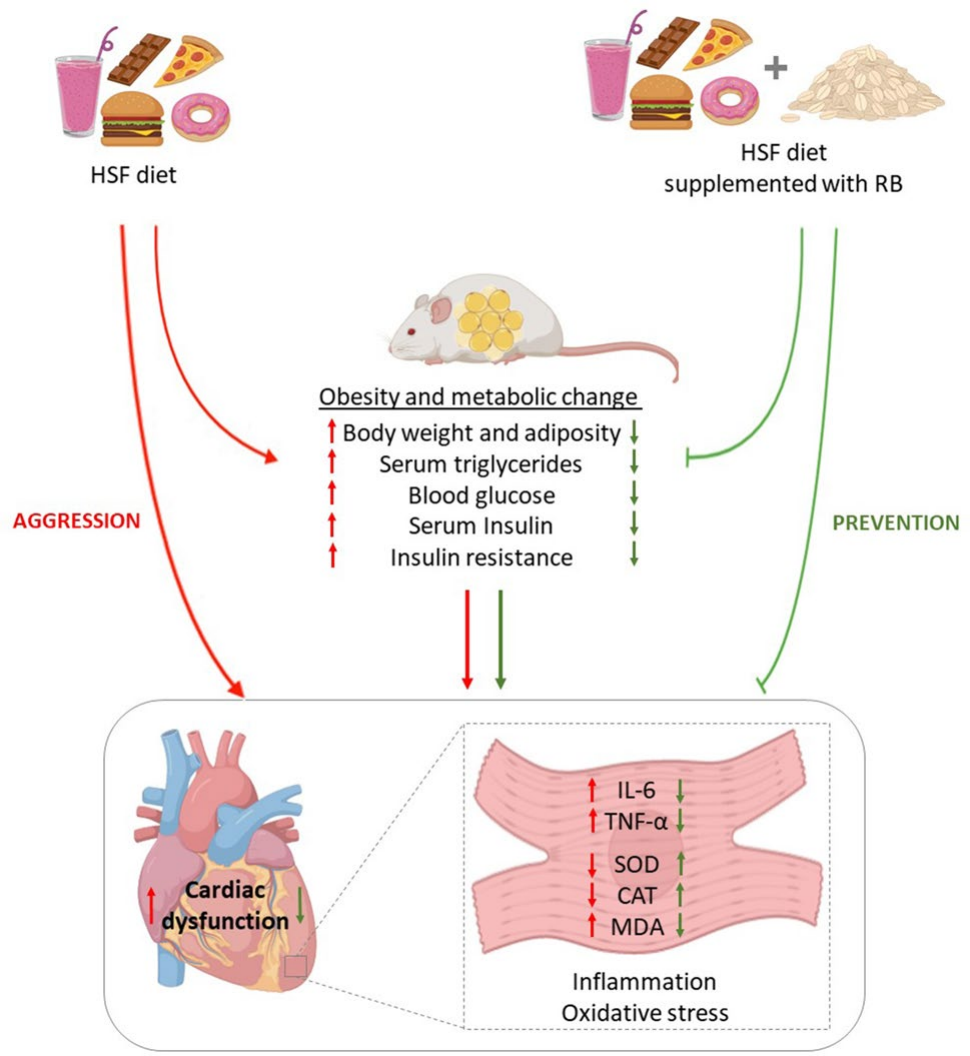
Overview of the changes induced by HSF diet (red arrows) and preventive effect of RB supplementation (green arrows). The HSF intake increases body weight and adiposity (obesity), leads to metabolic changes, cardiac dysfunction, increase of IL-6 and TNF-α levels in cardiac tissue and redox imbalance. The RB supplementation was able to prevent the metabolic changes, cardiac dysfunction, increase of cytokines and redox imbalance in cardiac tissue. *HSF* high-sugar fat diet, *RB* rice bran, *IL-6* interleukin-6; *TNF-α* tumoral necrosis factor; *SOD* superoxide dismutase; *CAT* catalase and *MDA* malondialdehyde

The dietary pattern high in saturated fat and sugars, known as the Western diet, dramatically influences the body composition, the development of metabolic complications, and cardiovascular diseases (CVDs). CVDs represents 17.9 million deaths annually [36]. Therefore, it is crucial to investigate alternatives to mitigate the consequences of excess adipose tissue, mainly non-pharmacological prevention strategies. The incorporation of natural foods into the diet with beneficial effects has been extensively evaluated as an alternative. The expressive amount of bioactive compounds presents in plant foods and their synergistic effect can act as a preventive agent [37]. In this context, this study aimed to evaluate the effect of RB supplementation in a high-sugar fat diet on cardiac dysfunction in an experimental obesity model. Our results revealed that dietary RB supplementation promoted amelioration of systemic metabolic complications, cardiac dysfunction, and inflammation and redox imbalance in the myocardium in a diet-induced obesity model (Fig. 7).

In a condition of positive energy balance, hypertrophy of adipocytes and hyperplasia of preadipocytes occur to store the energy in the form of triglycerides [38]. Although these processes are physiologically necessary, hypertrophy is mainly associated with adipose tissue dysfunction, resulting in adipocyte inability to store triglycerides and imbalance of adipokine secretion, leading to excessive production of reactive oxygen species (ROS) and pro-inflammatory cytokines. These factors lead to insulin resistance, redox imbalance, and chronic inflammation, which serve as a trigger for developing metabolic and cardiovascular complications [38–40]. Our findings showed that the rats that received RB supplementation did not accumulate fat and increase their body weight in the same proportion as the HSF group. This fact probably attenuated the adipose tissue role in the complications. Previous studies attributed some mechanisms, which may be involved in the anti-obesogenic and antilipidemic effect of RB. Firstly, one plausive explanation would be the ability of RB to provide a more significant elimination of fat in the feces due to the fiber content and the γ-oryzanol [41]. Another factor is its effect on lipid metabolism, preventing fat accumulation and decreasing body weight and adiposity index. Moreover, RB modulates gut microbiota decreasing the Firmicutes/Bacteroidetes ratio and the adipose tissue expression of uncoupling protein 1 (UCP1), peroxisome proliferator-activated receptor gamma coactivator 1-alpha (PGC 1-α), and PR domain containing 16 (PRDM16), as well as increase the expression of peroxisome proliferator-activated receptor gamma (PPAR-γ), favoring adipocyte hyperplasia and relieving the lipid overload triggered by hypertrophy [18, 42, 43].

The molecular mechanisms for the development of obesity-related glucose metabolism have also been researched [44]. Classic scientific evidence reports that inflammation due to adipose tissue expansion influences the intracellular pathway of insulin, causing damage in the translocation of GLUT4 to the plasma membrane by infusing the phosphorylation of insulin receptor substrates [45, 46]. This information agrees with our results since the animals that consumed HSF + BR stored 45% less body fat compared to the HSF group and possibly developed a lower hypertrophy degree and inflammation, which did not affect the glucose and insulin levels, thus not resulting in resistance to this hormone (Fig. 2B–D).

The diet-induced obesity model, as expected, presented relevant disruption in the cardiovascular system by developing hypertension, hypertrophy, and diastolic and systolic dysfunction. It is well known that obesity is associated with the cardiac remodeling in both humans and animal models, and several mechanisms are attributed to the functional impairment of the heart [47–50]. Our observations suggest the potential role of the inflammation and oxidative stress since HSF animals showed high levels of pro-inflammatory cytokines and an imbalance between antioxidant and pro-oxidant agents in cardiac tissue, which have been correlated with various parameters of cardiac function and structure (Fig. 5), corroborating previous studies [51]. Interestingly, RB supplementation prevented the development of structural and functional alteration and attenuated the pro-inflammatory and oxidative state in the heart of animals under HSF. In the literature, there are no studies that evaluated preventive RB supplementation on cardiac structure and function changes, except a work realized by our group [21], which showed that isolated γ-oryzanol significantly prevented structural and functional cardiac dysfunction in diet-induced obese animals for 20 weeks, but no via mechanistic target was evaluated. Here, our data suggested that RB was able to prevent the structural and functional cardiac impairment in obese animals, probably due to the mitigation of inflammatory and oxidative pathways. RB is comprised of a considerable number of phytochemicals with anti-inflammatory and antioxidant properties; among them, the γ-oryzanol is 13–20 times more in concentration compared to that of total tocotrienols and tocopherols in RB. Thus, it has gained special attention to their potential health benefits. The oil present in RB has been called the “Heart oil” since its chemical profile is considered as cardiac protective. Furthermore, the RB effect is associated with γ-oryzanol and not only with the fibers present in brans composition [14].

In relation to cardiac inflammation, we showed that obese rats supplemented with RB had lower cardiac IL-6 and TNF-α cytokines than obese animals without supplementation. Rao et al. [19] revealed that dietary γ-oryzanol of RB oil reduced pro-inflammatory mediators (IL-6 and TNF-α) secreted by peritoneal macrophages of rats. Also, in other studies, serum TNF-α level was decreased in obese mice fed a high-fat diet supplemented with RB compared to obese mice [52], and γ-oryzanol significantly reduced the up-regulated expression of IL-1β, IL-6, TNF-α, and COX-2 mRNA in mice with colitis [53]. The mechanisms by which RB or its compounds decreases pro-inflammatory cytokine production are not yet fully understood and have been less studied. However, evidence has been pointed out for the role in decreasing levels of Toll-like Receptors (TLR-2 and TLR-4) and Nuclear Factor-kappa B (NF-κB) [19, 52–54] and up-regulating adiponectin expression, an important anti-inflammatory marker [19].

The cardiac oxidative stress present in obese animals fed the HSF diet was affected by the RB supplementation inducing the rise in antioxidant enzymes, SOD and CAT, which may have influenced the decrease in MDA levels, ameliorating the pro-oxidant status in these animals. SOD, CAT, and GPx are the first line of enzyme-based cellular defense systems, and each enzyme displays a distinct role in the mitigation of oxidative injury, mainly combating ROS [55]. Investigations have shown that RB or their products and isolated compounds exert antioxidant properties by increasing the expression or activity of these antioxidant enzymes. Treatment with γ-oryzanol showed a significant protective antioxidant effect against oxidative stress in the glaucoma model for raising the down-regulated SOD, CAT, and GPx levels in this model [56]. The antioxidant cytoprotection effect of RB extract was also determined in rat H9c2(2–1) cardiomyocytes; the authors showed that RB elevated the enzymatic activity and gene expression of CAT [57]. The exact mechanism that allows these natural compounds to increase both expression and activity of antioxidant enzymes remains unclear; however, some studies evidence the association of this effect with nuclear factor erythroid 2-related factor 2 (Nrf2) pathway modulation [58]. Moreover, another antioxidant mechanism has also been proposed for the bioactive components of RB, e.g., their chemical structure that exhibit radical-scavenging activity [59].

Despite the promising results, limitations of this study include the lack of a deeper basic investigation to elucidate the molecular mechanisms and to confirm the potential pathways involved in the protective role of RB against cardiac dysfunction in obesity as discussed above. Additional research should better explore the underlying mechanisms of this effects so that clinical applications can be consider in the future.

Considering γ-oryzanol as the main compound in RB and its well-demonstrated antioxidant and anti-inflammatory activity, our results could be related to this. However, it is worth mentioning that the beneficial effects of RB supplementation on the heart, in addition to the possible direct action of its compounds, may also be due to the improvement of metabolic systemic parameters and reduction of body weight and fat.

Finally, we can emphasize that healthy eating habits are the primary step to prevent or to go along with medical treatment. However, the change of dietary pattern generates resistance from individuals; thus, reinforcing the inclusion of natural compounds in the usual diet, even before excluding harmful foods, already shows a positive result on health. Previous studies have shown dose translocation from experimental models to humans. In the present study, the RB dose in the diet was 11% (w/w), and the average daily food intake (HSF + RB group) was 11.0 g/day/animal, which corresponds to 1.21 g/day of RB. This is equivalent to 2.4 g/day/kg, considering the average weight of the animals (0.509 kg). According to the formula in the literature, which considers the body surface area, the corresponding dose of RB is 31 g/day for a human of 60 kg (0.52 g/day/kg) [60–62]. The 31 g of RB is equivalent to 6 soup-spoons measurements, representing an amount that can be easily introduced in human consumption.

In conclusion, this investigation provides evidence that RB supplementation prevented cardiac dysfunction by modulating systemic metabolic complications and the inflammation and oxidative stress in the myocardium of obese rats fed HSF, suggesting that the regular consumption of RB could be an alternative dietary approach to combat obesity-related complications. Further studies are required to confirm the beneficial effect on humans in clinical trials involving large cohorts and longitudinal follow-up.
